# Overcoming Alpine Barriers: The First Documented Case of Golden Jackal (*Canis aureus*) Settlement in a Subalpine Environment

**DOI:** 10.1002/ece3.74511

**Published:** 2026-10-23

**Authors:** Jonas Custers, Jennifer Hatlauf, Ancolie de Bergeyck, Jaka Črtalič, Boštjan Pokorny, Hubert Potočnik

**Affiliations:** ^1^ Institute of Wildlife Biology and Game Management, Department of Ecosystem Management, Climate and Biodiversity BOKU University Vienna Austria; ^2^ Independent Researcher Burdinne Belgium; ^3^ Department of Biology, Biotechnical Faculty University of Ljubljana Ljubljana Slovenia; ^4^ Faculty of Environmental Protection Velenje Slovenia; ^5^ Slovenian Forestry Institute Ljubljana Slovenia

**Keywords:** Alpine ecology, camera trapping, conservation biology, spatial analysis, wildlife dispersal

## Abstract

The golden jackal ( *Canis aureus* ) continues to expand across Europe, yet fine‐scale behavioural data on dispersal—particularly in mountainous regions—remain scarce. Reproducing individuals are recorded further and further to the North and West of the continent, crossing major anthropogenic and natural barriers in the landscape and settling within new habitats. Since jackals are already present or at the doorstep of almost every country on the European mainland, monitoring this range expansion is of great interest to the public and policymakers. The dispersal capacity of jackals is pivotal for their current expansion across Europe. However, research on their movement patterns, particularly natal dispersal, remains limited. Here, we analysed the full dispersal trajectory of a GPS‐collared subadult male originating from the Julian Alps (Slovenia). Using T‐LoCoH utilisation distributions, Net Squared Displacement modelling and Bayesian movement models, we reconstructed its pre‐dispersal behaviour, dispersal phase and subsequent settlement. After several initial exploratory excursions, the young male left its natal home range in mid‐November 2023 and travelled a cumulative distance of 170 km (96 km straight‐line distance), overcoming natural and anthropogenic barriers along the way. After 11 days, it established a stable home range in the Austrian Malta Valley, where reproduction was later confirmed in spring 2024—the first documented case of successful settlement and breeding by a golden jackal in a subalpine environment. During dispersal, the jackal's step length increased in areas with a higher aggregation index as well as at night, suggesting more extensive movement between consecutive GPS acquisitions in more homogeneous areas and during nighttime. Our study provides the first fine‐scale GPS documentation of dispersal, settlement, and subsequent reproduction of a golden jackal in a European subalpine environment. It highlights how both natural and human‐dominated landscapes shape movement behaviour during range expansion.

## Graphical abstract

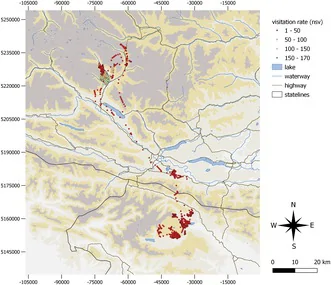

## 1 Introduction

The golden jackal ( *Canis aureus* ) is undergoing a significant range expansion into new regions of northern and western Europe (Hatlauf 2025; Krofel et al. 2017; Spassov and Acosta‐Pankov 2019; Stefanović et al. 2024; Trouwborst et al. 2015). While the species has historically been associated with lowland and semi‐open landscapes, its expansion challenges traditional assumptions about the ecological constraints that limit mesopredator distribution and highlights its adaptability to novel environmental conditions (Šálek et al. 2014; Heptner and Naumov 1974). For example, records of golden jackals have been increasing in European Subarctic and Arctic regions, where they have adapted by relying closely on anthropogenic food sources during harsh winters (Kojola et al. 2024; Oparin et al. 2024; Sørensen and Lindsø 2021; Rykov et al. 2022). To the west, golden jackals have reached as far as the Iberian Peninsula, for which they have had to cross the Pyrenees mountain range (Sáenz de Buruaga et al. 2023).

Mounting evidence now demonstrates that golden jackals can disperse into and, in some cases, establish populations in subalpine and alpine environments. In Bosnia and Herzegovina, Trbojević et al. (2018) have reported the presence of territorial groups at altitudes of at least 760 m a.s.l., and hunting records indicate jackals occurring as high as 1203 m a.s.l. Similarly, in Bulgaria, golden jackals have reportedly been killed by hunters in an area at the foot of the eastern Balkan Mountains at elevations ranging between 500 m to 1200 m a.s.l (Raichev et al. 2013). Later, Kirilov and Peeva (2021) confirmed jackal presence at 1000–1200 m a.s.l. in the same region. Moreover, in Greece, acoustic surveys confirmed sporadic golden jackal presence at altitudes up to 1050 m a.s.l. (Giannatos et al. 2005). In the Alps and the Dinaric Mountains, observations have also been reported at higher altitudes: in the Italian Alps, a territorial group was recorded at approximately 1800 m a.s.l. (Lapini et al. 2016), and in April 2019 an individual was photographed in the snow at an elevation of 2350 m a.s.l. (by D. Colombo). Dispersing individuals may reach very high altitudes while moving from one area to another; for example, a young male fitted with a GPS collar reached a maximum of 1498 m a.s.l. during its dispersal from the Slovenian Karst to northern Dalmatia, Croatia (Potočnik et al. 2019).

In the Himalayas, golden jackals have been reported at altitudes that significantly exceed the aforementioned altitude records. In the Greater Himalaya of the Indian Himalayan Region, records reach up to 2000 m (Hoffmann et al. 2018). Meanwhile, the species has been observed in the Manaslu Conservation Area of Nepal at an elevation of 3300 m a.s.l. (Katuwal and Dahal 2013), and at up to 3400 m a.s.l. in the alpine environment of Jilong County in Tibet (Dong et al. 2019). Khan et al. (2024) recently reported golden jackal presence in different areas of the Trans‐Himalayan region of Ladakh at altitudes ranging from 3120 up to 5365 m a.s.l., marking the highest known elevations recorded for golden jackals across their range known to date.

Climate change has been identified as one of the main drivers of the rapid expansion of the golden jackal in Europe, as rising temperatures are creating new suitable habitats in northern and western Europe (Flores‐Manzanero et al. 2026). Other ecological factors have also been reported to influence the golden jackal's presence and expansion in mountainous and high‐altitude areas, including the topographical landscape, vegetation types, anthropogenic activities (Fenton et al. 2021; Frangini et al. 2022; Pantelić et al. 2024; Serva et al. 2023; Spassov and Acosta‐Pankov 2019; Torretta et al. 2020) and the distribution and population density of the grey wolf (Krofel et al. 2017; Ranc et al. 2026). Golden jackals live in family groups consisting of a dominant adult pair and one or two generations of their offspring (Custers et al. 2024; Macdonald 1979). After reaching sexual maturity at approximately 11 months of age, young golden jackals leave their natal range to establish a new family group elsewhere in a process called dispersal (Lanszki et al. 2018).

Dispersal can be defined as the movement leading to spatial gene flow, typically followed by reproduction. The dispersal process consists of three different stages: emigration, movement and immigration into a new patch (Clobert et al. 2012). Although dispersal has been the focus of extensive empirical and theoretical investigation (Chaine et al. 2013; Clobert et al. 2012), GPS‐tracked dispersal trajectories remain rare across species (e.g., Morales‐González et al. 2022). Nevertheless, GPS telemetry has provided important new insights into golden jackal dispersal, despite limited reporting. For instance, Lanszki et al. (2018) were the first to document the dispersal event of a yearling female golden jackal, which covered approximately 96 km in linear distance over approximately 12 days, after which she settled and had reproductive success. The total distance travelled during dispersal appears to be mainly affected by time of day, with both jackals showing a strong tendency towards nocturnal movement (Fenton et al. 2021; Lanszki et al. 2018; Pantelić et al. 2024). Dispersing golden jackals can travel great distances before settling, with recorded distances of up to 3400 km (Bogdanowicz et al. 2025; Lanszki et al. 2018; Potočnik et al. 2022). Although a difference between sexes in dispersal movement is commonly observed in both social and solitary carnivores (e.g., Herrero et al. 2021), there is no conclusive evidence that this is also the case for golden jackals, as both genetic data and telemetry studies fail to provide evidence to suggest that males can disperse over larger distances than females (Bogdanowicz et al. 2025; Lanszki et al. 2018; Stefanović et al. 2024). However, the first recorded jackal individuals in new areas have, in all cases so far, been males (Hatlauf and Krickl 2025).

In this study, we closely investigated the dispersal movement behaviour of an approximately one‐year‐old subadult male golden jackal, ‘Maj’, fitted with a GPS collar in a subalpine environment, defined here as the zone just below the treeline. Furthermore, we have documented the eventual settlement and family founding of the studied golden jackal. We hypothesised that dispersal behaviour would be strongly shaped by both anthropogenic and natural landscape features (Lanszki et al. 2018; Potočnik et al. 2022). Specifically, we predicted that (1) the golden jackal would increase step length at night and in human‐dominated areas (Fenton et al. 2021; Lanszki et al. 2018; Pantelić et al. 2024); (2) resting sites would be located in areas providing cover in proximity to water and food sources (Fenton et al. 2021; Frangini et al. 2022); (3) dispersal would be characterised by a distinct phase of strong spatial displacement followed by settlement; and (4) mountainous terrain would not impede displacement during dispersal.

By analysing the dispersal trajectory of this jackal and its eventual settlement, we aimed to understand how an individual can navigate and colonise subalpine landscapes. This case study provides a valuable, high‐resolution record of a complete dispersal event, contributing to our understanding of the dispersal process and the ongoing range expansion of golden jackals in Europe.

## 2 Materials and Methods

### 2.1 GPS Tracking

The male golden jackal was captured in June 2023 using a foothold‐activated trap deployed for wolf monitoring on the Pokljuka plateau in Slovenia. Upon capture, he was chemically immobilised with a solution of 100 mg/mL Ketamine HCl [A] and 1 mg/mL Medetomidine HCl [B] at a dosage of 0.05 mL/kg [A] + 0.05 mL/kg [B], administered intramuscularly using a hand syringe. Standard biometrics (sex, age estimation by dentition, mass, body length) were recorded. The animal was fitted with a Vectronic Aerospace GPS‐GSM collar (model Vertex Lite; weight = 2.3% of body mass). The collar was programmed for variable acquisition intervals (approx. 2–4 h) that were intentionally changed to optimise data collection (maximum gap length = 33.51 h, fix success rate = 97.9%), and its positional accuracy was validated at four static test points prior to deployment (mean horizontal error = 8.3 m). Total handling time was approximately 38 min, after which the individual received an antidote (atipamezole HCl), recovered, and was released at the capture location. All capture and handling procedures complied with national animal welfare legislation and were approved by the relevant authority (permit number 341‐159/2023/7).

### 2.2 Movement Analysis

Statistical analyses were conducted in R version 4.4.2 (R Core Team 2024) using RStudio version 2024.12.0 (Posit team 2024). GPS locations were irregularly spaced (median interval: 3.97 h). To enable time‐based movement analyses, we linearly interpolated GPS positions during all stages of dispersal at 4‐h intervals (number of raw fixes = 2475, number of interpolated fixes = 2471), with 95% of intervals ranging from 2.07 h (2.5th percentile) to 9.51 h (97.5th percentile). This ensured uniform sampling without introducing artificial bursts. Interpolation preserves displacement patterns at the scale of hours but may underestimate fine‐scale tortuosity; we therefore interpret cumulative distance as a conservative (minimum) estimate of the true distance travelled.

To estimate utilisation distributions and movement structure, we applied the Time Local Convex Hull (T‐LoCoH) framework, which is a method for constructing home ranges and exploring spatio‐temporal patterns in movement data (Lyons et al. 2013). The space–time scaling parameter (*s*) was optimised using the ptsh‐based method and set to 0.02 (Lyons 2014). We evaluated a sequence of *k* values (6–24 in steps of 3) and selected *k = 15* based on the stability of isopleth area curves and edge‐to‐area ratios (Lyons 2014).

We then calculated additional hull metrics to characterise the movement behaviour and space use of the golden jackal, following the workflow established by Lyons (2014). First, we determined hull elongation by constructing a bounding ellipse for each hull and computing its eccentricity. This metric reflects the degree of directional movement. Second, we determined time‐use metrics using an inter‐visit gap (IVG) of 12 h, a temporal scale appropriate for analysing the daily movement behaviour. The time‐use metrics quantify the number of separate visits (nsv) and the mean number of locations per visit (mnlv) for each hull. Next, to identify patterns in the golden jackal's movement behaviour, we constructed behavioural isopleths by sorting hulls by eccentricity and subsequently merging them into cumulative polygons. Afterwards, we calculated net squared displacement (NSD) over time as the squared Haversine distance from the golden jackal's first recorded location using the R package *geosphere* (Hijmans 2024). The NSD looks at the movement vectors of animals to determine their use of the landscape (Bunnefeld et al. 2011; Papworth et al. 2012) and can therefore be used to identify transitions between different stages of dispersal based on changes in displacement over time (Börger and Fryxell 2012; Singh et al. 2016).

Here, we calculated NSD as the squared straight‐line distance between the first GPS location and each subsequent location (Bunnefeld et al. 2011; Turchin 1998):

$${\mathrm{NSD}}_{i}=\text{distHaversine}{\left(\left({\mathrm{lon}}_{i},{\mathrm{lat}}_{i}\right),\left({\mathrm{lon}}_{0},{\mathrm{lat}}_{0}\right)\right)}^{2},$$

where $\left({\mathrm{lon}}_{0},{\mathrm{lat}}_{0}\right)$ is the first fix location. Next, we fitted its NSD trajectory over time with a logistic (sigmoid) model to describe the dispersal pattern (Bunnefeld et al. 2011):

$$\mathrm{NSD}\left(t\right)=\frac{\text{Asym}}{1+\exp\left(\;-\frac{t-\text{xmid}}{\text{scal}}\right)},$$

where ‘Asym’ is the asymptotic NSD (maximum displacement squared), ‘xmid’ is the time (in days) at which the value for displacement reaches half of ‘Asym’, and ‘scal’ is the parameter controlling the steepness of the curve. We used nonlinear least squares to estimate model parameters.

To validate the logistic NSD model, we applied non‐parametric LOESS smoothing to the displacement data. Both curves aligned closely, particularly during the mid‐ to late phase of the movement trajectory, indicating that the logistic model sufficiently captured the overall movement pattern of the golden jackal.

Furthermore, we estimated the onset and end dates of its dispersal process based on the asymptotic NSD. We defined dispersal onset as the first day on which NSD exceeded 2% of the asymptotic NSD, and dispersal end as the second time NSD reached the asymptotic NSD value with a two‐day stabilisation period. The 2% threshold was chosen to detect the earliest sustained deviation from the pre‐dispersal home range while avoiding classification of short exploratory excursions. Additional visualisations were prepared using the R package *moveVis* (Schwalb‐Willmann et al. 2020) and QGIS (QGIS Development Team 2025).

### 2.3 Resting‐Site and Landscape‐Use Analysis

To analyse resting‐site selection by the golden jackal, we followed a multi‐step workflow similar to that used by Torretta et al. (2023) in their study of wolf dispersal patterns and resting‐site selection.

Resting sites were identified by applying a 250 m buffer around each interpolated location during dispersal (120 locations used). A location was classified as a resting site when the golden jackal remained within overlapping buffers for ≥ 6 consecutive hours. The 250 m scale reflects typical fine‐scale movements around resting sites observed in canids and exceeds positional error (Torretta et al. 2023). For each cluster of overlapping buffers identified as a resting site, we calculated the centroid of the cluster and used its 250 m buffer as the representative resting‐site location.

Second, we calculated multiple environmental covariates (Table 1) for all interpolated GPS locations for subsequent modelling of resting‐site selection. These covariates included the most prevalent land‐cover class, categorised into four groups (‘anthropogenic’: CLC 112, 122; ‘agriculture’: CLC 231, 242, 243; ‘forest’: CLC 311, 312, 313; and ‘other’: all other CLCs; CORINE Land Cover 100 m, 2018), mean tree‐cover density within the 250 m buffer (HRL Tree Cover 10 m, 2021), mean human settlement density within the 250 m buffer (GHSL 100 m, 2025; Pesaresi et al. 2025), minimum distances from roads (as a proxy for accessibility and disturbance by traffic) and rivers (as a proxy for proximity to water) to the centroid of each buffer (OpenStreetMap), minimum distances from tree cover (as a proxy for cover provided by tree canopies) and built‐up patches (as a proxy for proximity to anthropogenic environments) to the centroid of each buffer (OpenStreetMap), the mean slope within each 250 m buffer (Copernicus Digital Elevation Model), time of day, and GPS‐derived altitudes were considered. Additionally, we considered more landscape metrics within a 250 buffer around each interpolated datapoint (Torretta et al. 2023), which we calculated using the R package *landscapemetrics* (based on CORINE Land Cover 100 m, 2018) (Hesselbarth et al. 2019).

**TABLE 1:** Environmental covariates extracted for the interpolated GPS locations and used for the modelling of resting‐site selection of the studied golden jackal.

Variable	Description
mean_altitude	Mean distribution of the altitude (m)
**CLC_CODE** ^**a**^	Corine landuse class category (urban, agricultural, natural)
**mean_TCD** ^**b**^	Mean distribution of the tree cover density (%)
**mean_GHSL** ^**c**^	Mean distribution of the human settlement density (%)
**min_distance_to_road_m** ^**d**^	Minimum distance from roads (m), representing proximity to traffic and associated anthropogenic disturbance
**min_distance_to_river_m** ^**d**^	Minimum distance from rivers (m), representing proximity to waterways
**ai_mean** ^**e**^	Mean distribution of the aggregation index (%)
area_mn_mean^e^	Mean distribution of the patch area (m^2^)
np_sum^e^	Number of patches
pd_mean^e^	Mean distribution of the patch density (/100 ha)
pr_sum^e^	Patch richness
prd_mean^e^	Patch richness density (/100 ha)
**mean_slope** ^**f**^	Mean distribution of the slope (%)
**dist_to_veg_m** ^**d**^	Mean distribution of the distance from tree patches (m), representing the availability of vegetative cover
**dist_to_building_m** ^**d**^	Mean distribution of the distance from building patches (m), representing proximity to anthropogenic environments and associated disturbance
**time_of_day**	Time of day (day for 7:00–17:00, night for 17:00–7:00)

*Note:* Variables in bold are included in further analyses. Other variables were excluded due to correlations.

^a^ European Union's Copernicus Land Monitoring Service Information (2020).

^b^ European Union's Copernicus Land Monitoring Service Information (2024).

^c^ Pesaresi et al. (2024).

^d^ OpenStreetMap contributors (2017).

^e^ Hesselbarth et al. (2019).

^f^ European Space Agency (2024).

After calculating these covariates, we converted distance values to exponential decays, running from 0 (distance = 0) to 1 (large distances) (Torretta et al. 2023). Additionally, we standardised all other continuous variables using *z*‐score normalisation with mean = 0 and SD = 1. We checked for correlations and dropped highly correlated covariates (abs(*ρ*) > 0.5) accordingly from the model predictors. In total, eight covariates remained (mean_TCD, mean_GHSL, ai_mean, mean_slope, min_distance_to_road_scaled, min_distance_to_river_scaled, dist_to_veg_scaled, dist_to_building_scaled). For continuous predictors, we assessed the linearity on the logit scale using the conditional effects plots. None of these predictors showed strong deviations from linearity; therefore, we retained all of them as linear terms.

The remaining environmental covariates were used in three Bayesian GLMs (Table 1). We modelled (1) resting‐site selection using a Bernoulli likelihood and logit link, (2) step length using a first‐order autoregressive (AR(1)) temporal correlation structure and (3) turning angle using gamma regression with a log link. Non‐informative (‘flat’) priors were used for all parameters. Consequently, the probability that a specific interpolated GPS location at time *i* would be selected as a resting site can be represented by the following equation:

$$Y_{i}\sim \text{Bernoulli}\left(p_{i}\right),$$

with

$$p_{i}=P\left(Y_{i}=1|x_{i}\right)=E\left[Y_{i}=1|x_{i}\right]$$

and

$$\text{logit}\left(p_{i}\right)=\beta_{0}+\beta_{1}X_{1i}+\cdots +\beta_{k}X_{\mathrm{ki}}.$$

where each $X_{i}$ is a predictor and each $\beta_{i}$ is the regression coefficient.

In this model, the effect of each variable is quantified by a single parameter, alpha (α), which is estimated on the logit scale. Therefore, to quantify the true effect sizes, we transformed the effects to the odds scale.

Additionally, we modelled step length during dispersal using lognormal regression after confirming the approximate normality of log‐transformed step lengths via Q–Q plots and posterior predictive checks. In this model, the response variable *y* represents the step length between two intervals. We used a logarithmic link function with a normally distributed error structure on the log scale. We included an AR(1) correlation structure to account for temporal autocorrelation. This adequately removed the correlation, as confirmed by inspecting the residual autocorrelation functions.

Here, step length can be represented by the following equation:

$$\log\left(Y\right)\sim \text{Normal}\left(\mu ,\sigma \right),$$

with

$$\log\left(Y_{t}\right)=\beta_{0}+\beta_{1}X_{1t}+\cdots +\beta_{k}X_{\mathrm{kt}}+\epsilon_{t}$$

and

$$\mu =\beta_{0}+\beta_{1}X_{1}+\cdots +\beta_{k}X_{k},$$

where each $X_{i}$ is a predictor and each $\beta_{i}$ is the regression coefficient.

Finally, we modelled the absolute values of the turning angles (bounded by 0 and π) during the dispersal process using gamma regression. Here, the response variable *y* represents the turning angle at each 2‐h interval. We used a logarithmic link function with a gamma error structure.

The turning angle can be represented by the following formula:

$$Y_{i}\sim \text{Gamma}\left(\mu_{i},\varphi \right),$$

where

$$\log\left(\mu_{i}\right)=\beta_{0}+\beta_{1}X_{1i}+\cdots +\beta_{k}X_{\mathrm{ki}},$$

and φ denotes the precision parameter of the Gamma distribution.

For each model, we obtained parameter estimates via Bayesian inference using Markov chain Monte Carlo (MCMC) methods in the R package *brms* (Bürkner 2017). We considered posterior effects as ‘considerable’ when the 95% Highest Posterior Density Interval (HPDI) excluded zero (on the logarithmic scale).

For all three models, we ran three chains across three computer cores, with 4000 iterations per chain and a warm‐up phase of 1000 iterations. Residuals from the models with continuous responses (step length and turning angle) did not show substantial remaining autocorrelation at lag 1 or higher (ACF plots, see Figure S1). We visually inspected the trace plots for each model parameter to confirm that chain convergence was achieved with these fixed iterations across the three chains. We reconfirmed chain convergence after checking the trace plots from the same model with twice the number of warm‐up and post‐warm‐up iterations (30,000 iterations in total). Next, we confirmed that the *R̂* (*R*‐hat) convergence diagnostic for each variable was below 1.01, indicating adequate mixing of chains (Vehtari et al. 2021). Estimated bulk effective sample sizes (bulk‐ESS) and tail effective sample sizes (tail‐ESS) were at least 100 (approximately) per Markov chain for all variables, indicating that estimates of respective posterior quantiles are reliable (Vehtari et al. 2021). Furthermore, all points of the WAMBS checklist (Depaoli and van de Schoot 2017) were addressed to assess the model's reliability (for a complete overview of the checklist points applied to the model, see Data S1–, S3).

In addition, to visualise resting‐site selection, step length, and turning angle throughout the dispersal process, we constructed three plots displaying the elevation gain of the golden jackal over the travelled distance (Figure S2).

## 3 Results

Following capture and GPS‐collaring in June 2023, the golden jackal established a transient home range on the pre‐Alpine Pokljuka plateau in the Julian Alps (Slovenia), where it remained until mid‐November. Short‐distance relocations and spatial clustering characterised its movement patterns during this period, indicating exploratory behaviour. However, in November 2023, a marked shift towards linear, longer‐distance travel occurred, signalling the onset of dispersal (overview in Figure 1).

**FIGURE 1:**
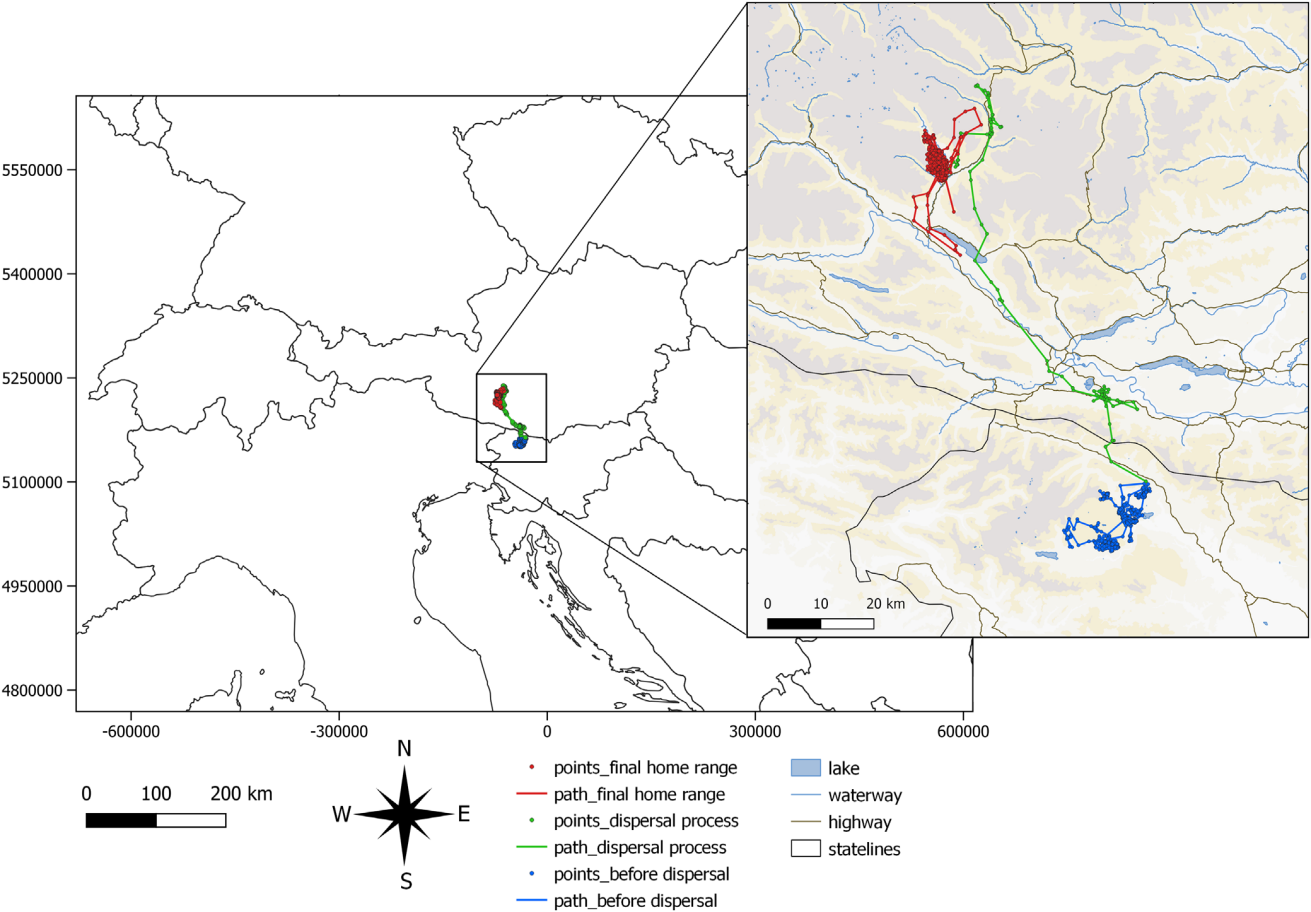
Map illustrating the golden jackal's movement trajectory from the Julian Alps (Slovenia) to Carinthia (Austria).

The jackal initiated dispersal on 14 November 2023 (Day 154 post‐capture). It travelled northward into Austria. Based on interpolated locations at 4‐h intervals, the cumulative minimum distance travelled during dispersal was 170 km. This value represents a conservative estimate and does not capture fine‐scale tortuosity. Meanwhile, the net straight‐line displacement between the origin and the final settlement area was approximately 75 km. The individual crossed multiple major anthropogenic structures (at least 20 highway crossings) and natural barriers, including elevations up to 1950 m a.s.l. The maximum straight‐line distance from its pre‐dispersal home range was 96 km, recorded at the Katschberg ski resort in Austria. This distance exceeded the net displacement between origin and settlement area (75 km) because the individual temporarily moved beyond its eventual settlement location before establishing a territory.

By late November 2023, the individual established a stable home range on the northern slopes at the entrance to the Malta Valley. Over the winter, its spatial behaviour shifted to a more localised pattern, suggesting the establishment of a permanent territory. Despite three exploratory excursions lasting 2–4 days in February 2024, during the mating season, no evidence of further dispersal or excursions was recorded. Monitoring ended on 5 August 2024 following loss of the GPS signal. The reason could not be determined, although mortality is considered a possible explanation.

### 3.1 Movement Analysis

Mapping the isopleths (Figure 2) and plotting the corresponding area curves (Figure S3a) and edge‐to‐area ratios (Figure S3b) confirmed that the estimated home ranges were stable. Consequently, we used the same hull set for all subsequent analyses. The 95% isopleths representing the total home‐range extent before and after dispersal had an area of 25.42 and 23.34 km^2^, respectively.

**FIGURE 2:**
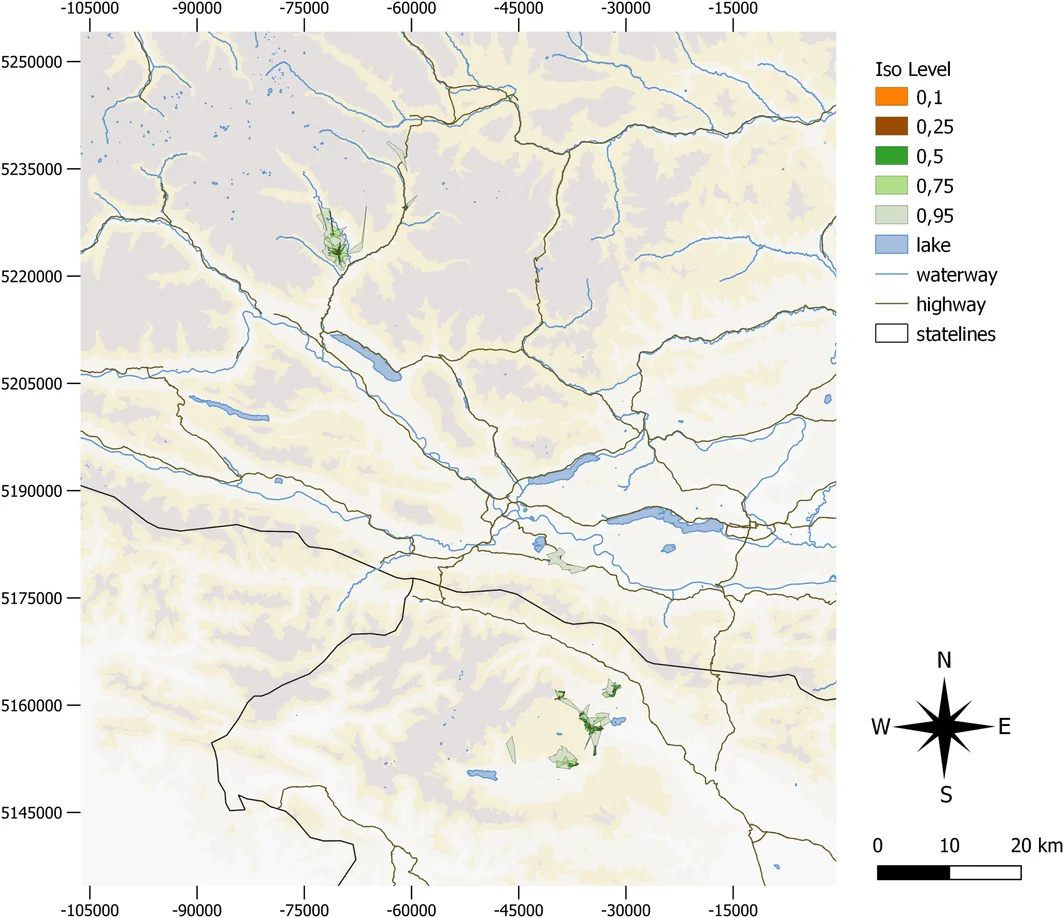
Map illustrating the isopleths created for the hull sets determined for the golden jackal's movement trajectory with *k* = 15, moving from the Julian Alps (Slovenia) to Carinthia (Austria).

Hull elongation values exhibited high eccentricity values during the dispersal period, indicating strong directional bias (Figure 3). In contrast, eccentricity values were lower during the pre‐ and post‐dispersal period, indicating low directionality. Time‐use metrics we determined based on a 12‐h inter‐visitation gap (IVG) showed that the individual frequently revisited certain locations (Figure 4).

**FIGURE 3:**
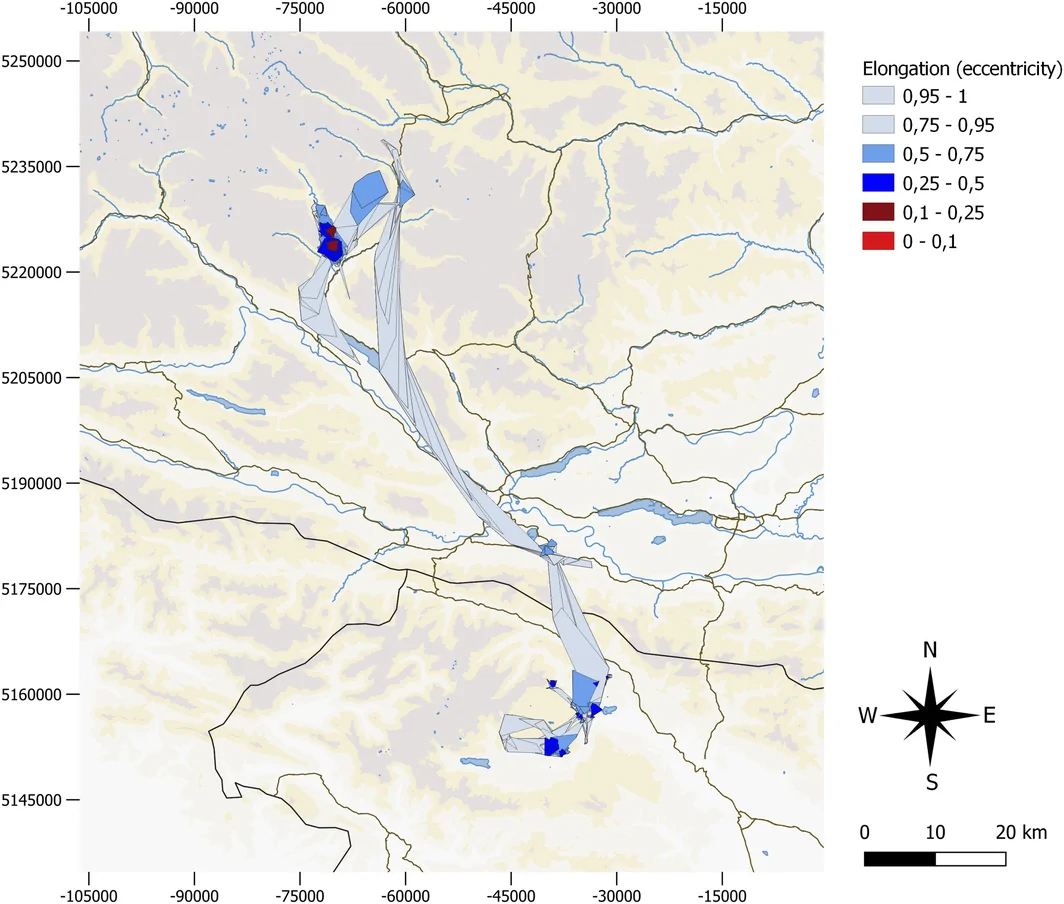
Map illustrating the hull elongation for the hulls determined for the golden jackal's movement trajectory with *k* = 15, moving from the Julian Alps (Slovenia) to Carinthia (Austria).

**FIGURE 4:**
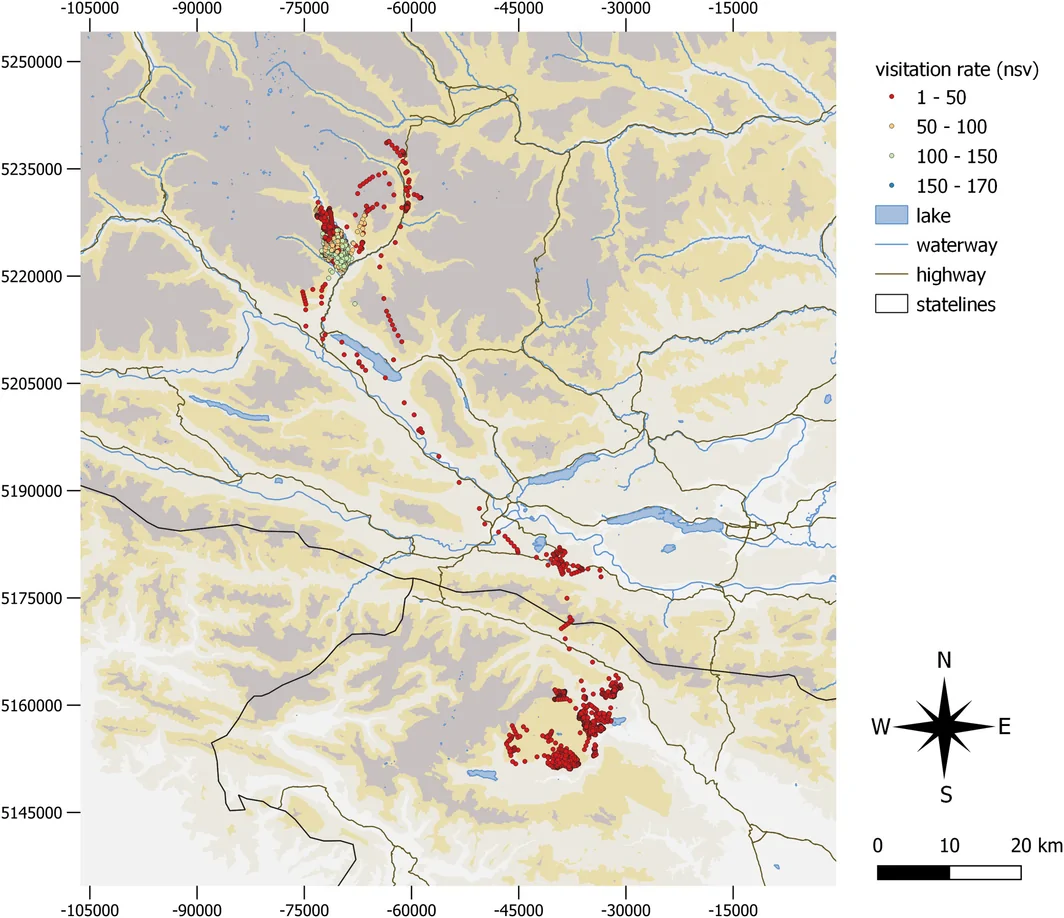
Map illustrating the revisitation rate (nsv) for the golden jackal's movement trajectory with *k* = 15, moving from the Julian Alps (Slovenia) to Carinthia (Austria).

Analysis of the net squared displacement (NSD) trajectory, using the predefined 2% threshold, revealed the onset of dispersal at approximately 154 days after capturing (14 November 2023). We determined the end of the dispersal process at approximately 165 days after capturing (3 December 2023). Although the interval between the first and last dispersal‐related movements spanned approximately 19 calendar days, the NSD‐based dispersal phase itself lasted approximately 11 days. These 11 days represent the period during which the jackal exhibited sustained net displacement from its pre‐dispersal range, which is consistent with the main dispersal process. Meanwhile, the remaining eight days include movements that did not result in sustained net displacement from the original home range, such as exploratory movements and temporary excursions.

The logistic (sigmoid) model fitted to the NSD trajectory described the displacement pattern well. There was a relatively clear transition from pre‐dispersal to dispersal and from dispersal to post‐dispersal (Figure 5). The estimated value for asymptotic NSD (*Asym*), representing the maximum net displacement predicted by the model, was 4.91 × 10^9^ m^2^. The half‐displacement time (*xmid*), or the point where the jackal reached half of its eventual net displacement, was 156 days. The steepness parameter (*scal*) was 0.54 per days. This relatively steep logistic transition represents a relatively fast increase of the jackal's net displacement around the midpoint of the fitted NSD trajectory. These results support our prediction that directional, high‐eccentricity movement would characterise the jackal's dispersal phase. Nevertheless, the observed NSD occasionally exceeded the model‐predicted asymptotic displacement, particularly during the latter part of dispersal and subsequent excursions. These short rises in NSD, showing that the jackal's movements were not strictly uni‐directional, were not captured by the logistic fit because it assumes a monotonic approach towards the asymptote. They represent temporary excursions beyond the main settlement area.

**FIGURE 5:**
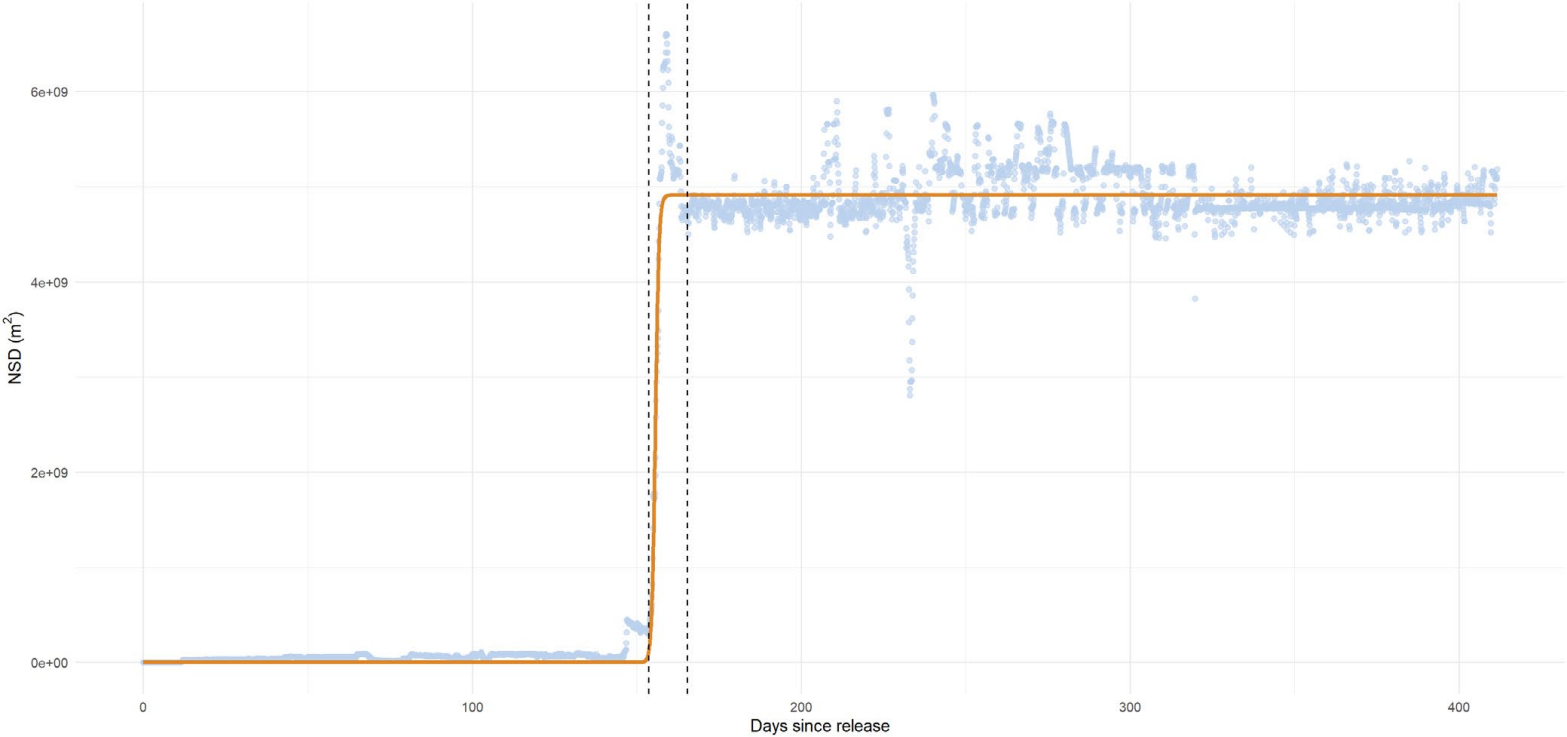
Observed and modelled net squared displacement (NSD) for the golden jackal's movement trajectory. Blue points represent observed NSD values over time (days since release), and the orange line shows the fitted logistic (sigmoid) model. Dashed vertical lines indicate the estimated onset (left) and end (right) of dispersal.

### 3.2 Landscape‐Use Analysis (Bayesian Modelling)

#### 3.2.1 Resting Site Selection

The posterior model estimates indicated that none of the environmental covariates had considerable effects on resting site selection, defined by the 250 m buffers around each interpolated GPS location in which the jackal stayed for more than 6 h (Table 2, Figure 6). These findings do not support our prediction that the jackal would select resting sites with more cover and structural fragmentation. Neither did we find any evidence that its selection of resting sites is affected by any of the other considered covariates.

**TABLE 2:** Means of the posterior samples for the variables used in the Bayesian logistic regression model with resting site as the response variable (on both the logit and odds scale).

Covariate	Description	Effect size (logit)	Effect size (odds)
Intercept	Model intercept	−1.96	0.14
		(−3.47, −0.58)	(0.03, 0.56)
CLC_groupOther	Effect of Corine Land‐use Class category that is neither nature, agriculture or anthropogenic	0.18	1.19
		(−1.68, 2.01)	(0.19, 7.50)
CLC_groupAgriculture	Effect of Agriculture as Corine Land‐use Class category	−0.41	0.66
		(−1.76, 0.91)	(0.17, 2.47)
CLC_groupAnthropogenic	Effect of Anthropogenic as Corine Land‐use Class category	0.84	2.31
		(−0.69, 2.41)	(0.50, 11.17)
mean_TCD	Effect of the mean HRL Tree Cover Density	0.87	2.38
		(−0.09, 1.86)	(0.91, 6.43)
mean_GHSL	Effect of the mean Global Human Settlement Density	−0.53	0.59
		(−1.77, 0.57)	(0.17, 1.76)
ai_mean	Effect of the mean aggregation index	−0.69	0.5
		(−1.50, 0.09)	(0.22, 1.10)
mean_slope	Effect of the mean slope	−0.12	0.88
		(−0.98, 0.68)	(0.37, 1.98)
time_of_daynight	Effect of night as the time of day	0.50	1.65
		(−0.95, 1.92)	(0.39, 6.83)
min_distance_to_road_scaled	Effect of the scaled minimum distance to roads	−0.31	0.74
		(−1.18, 0.47)	(0.31, 1.60)
min_distance_to_river_scaled	Effect of the scaled minimum distance to rivers	−0.08	0.93
		(−0.86, 0.72)	(0.42, 2.04)
dist_to_veg_scaled	Effect of the scaled minimum distance to plant cover	−0.66	0.52
		(−1.83, 0.40)	(0.16, 1.50)
dist_to_building_scaled	Effect of the scaled minimum distance to buildings	−0.51	0.60
		(−1.51, 0.36)	(0.22, 1.44)

*Note:* The 95% highest posterior density intervals (HPDIs) are shown in brackets. No covariate had a considerable effect.

**FIGURE 6:**
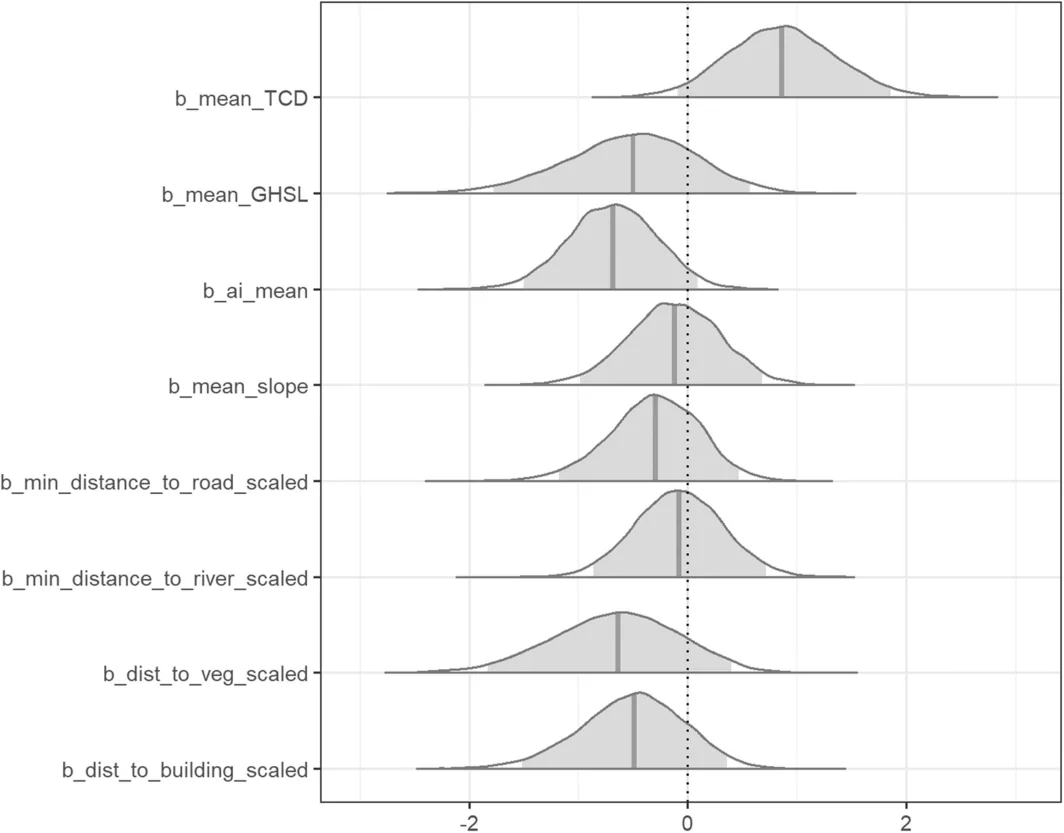
Posterior effects of the continuous predicting variables estimated by the Bayesian logistic regression model with resting site‐selection as the dependent variable. Model estimates (marked with a vertical bold grey line) and HPDIs (marked in grey) are presented on the logarithmic scale. Effects are considered considerable when the HPDI does not include zero.

#### 3.2.2 Step Length

The posterior estimates of the Bayesian lognormal model with step length as the response indicated two considerable positive effects on the logarithmic scale (Table 3). These included mean aggregation index (estimate = 0.39, 95% HPDI 0.05–0.73) and night as time of day (estimate = 1.15, 95% HPDI 0.52–1.77) (Table 3, Figure 7). These posterior effects confirm our prediction that the jackal would increase its step length at night. However, we also predicted that it would increase its step length in human‐dominated areas, and we did not find any evidence supporting this claim. Nevertheless, the posterior effects revealed a considerable effect for mean aggregation index. This shows that the jackal covered greater distances between consecutive GPS locations in areas with a higher degree of landscape homogeneity.

**TABLE 3:** Means of the posterior samples for the variables used in the Bayesian lognormal regression model with step length as the response variable (on the log scale).

Covariate	Description	Effect size (log)
Intercept	Model intercept	5.34
		(4.79, 5.88)
CLC_groupOther	Effect of Corine Land‐use Class category that is neither nature, agriculture or anthropogenic	−0.77
		(−0.77, 1.14)
CLC_groupAgriculture	Effect of Agriculture as Corine Land‐use Class category	0.86
		(−0.10, 1.84)
CLC_groupAnthropogenic	Effect of Anthropogenic as Corine Land‐use Class category	0.44
		(−1.58, 2.45)
mean_TCD	Effect of the mean HRL Tree Cover Density	−0.08
		(−0.44, 0.28)
mean_GHSL	Effect of the mean Global Human Settlement Density	−0.22
		(−0.57, 0.12)
ai_mean^a^	Effect of the mean aggregation index	0.39
		(0.05, 0.73)
mean_slope	Effect of the mean slope	0.17
		(−0.15, 0.50)
time_of_daynight^a^	Effect of night as the time of day	1.15
		(0.52, 1.77)
min_distance_to_road_scaled	Effect of the scaled minimum distance to roads	0.01
		(−0.28, 0.30)
min_distance_to_river_scaled	Effect of the scaled minimum distance to rivers	0.20
		(−0.09, 0.50)
dist_to_veg_scaled	Effect of the scaled minimum distance to plant cover	−0.06
		(−0.42, 0.29)
dist_to_building_scaled	Effect of the scaled minimum distance to buildings	0.16
		(−0.18, 0.48)

*Note:* The 95% highest posterior density intervals (HPDIs) are shown in brackets.

^a^ Variable with considerable effect.

**FIGURE 7:**
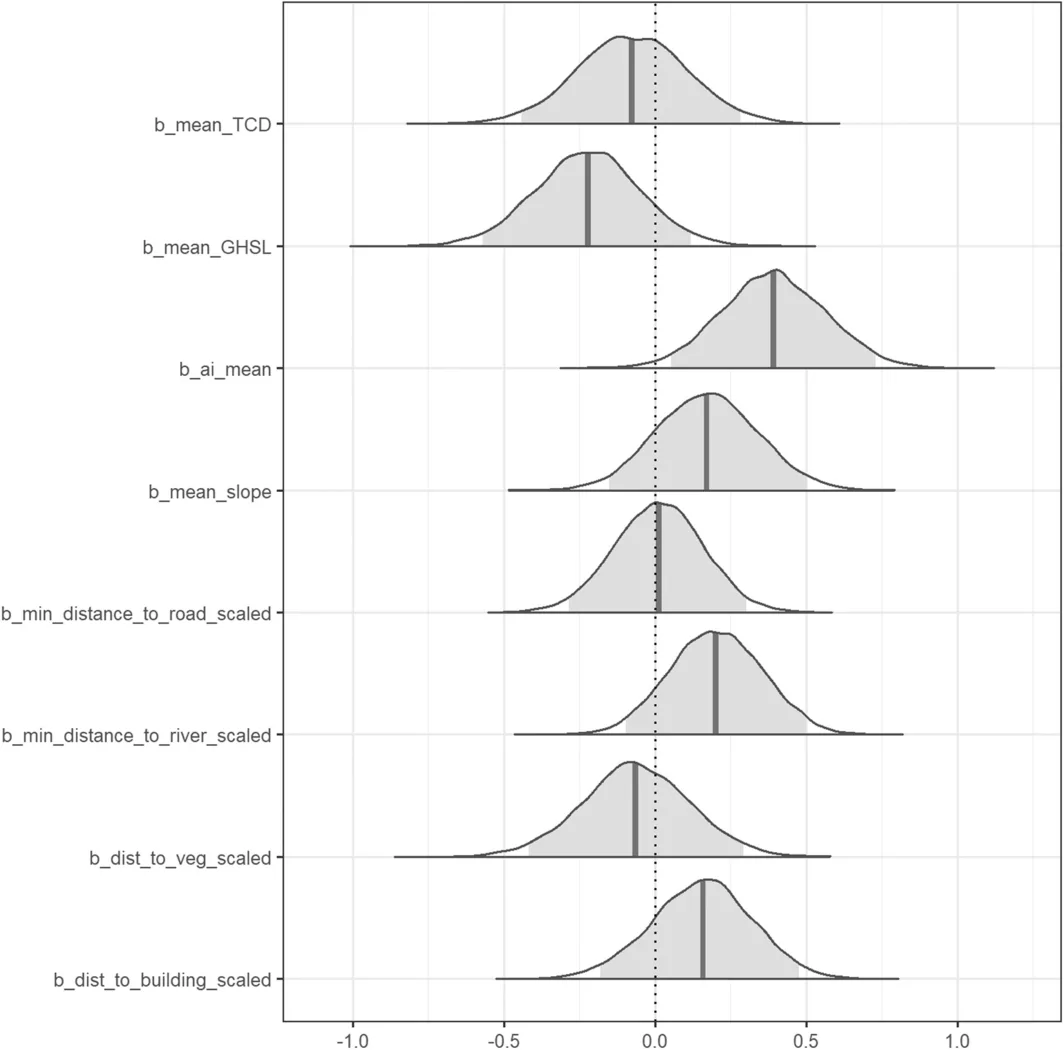
Posterior effects of the continuous predicting variables estimated by the Bayesian lognormal regression model with step length as the dependent variable. Model estimates (marked with a vertical bold grey line) and HPDIs (marked in grey) are presented on the logarithmic scale. Effects are considered considerable when the HPDI does not include zero.

#### 3.2.3 Turning Angle

The posterior estimates of the Bayesian gamma model with turning as the response did not reveal considerable effects of any of the environmental covariates (Table 4, Figure 8). Therefore, we did not find any clear evidence suggesting that the dispersing jackal's movement directionality and persistence are affected by any of the considered environmental covariates. Environmental covariates related to alpine terrain, such as altitude and slope, did not show clear effects on both step length and turning angle. Thus, these models provide no evidence that alpine terrain constrained the intensity or directionality of the jackal's movements during dispersal.

**TABLE 4:** Means of the posterior samples for the variables used in the Bayesian gamma regression model with turning angle as the response variable.

Covariate	Description	Effect size (log)
Intercept	Model intercept	1.29
		(−0.79, 1.86)
CLC_groupOther	Effect of Corine Land‐use Class category that is neither nature, agriculture or anthropogenic	0.25
		(−0.68, 1.23)
CLC_groupAgriculture	Effect of Agriculture as Corine Land‐use Class category	−0.20
		(−1.16, 0.78)
CLC_groupAnthropogenic	Effect of Anthropogenic as Corine Land‐use Class category	−0.90
		(−3.43, 1.87)
mean_TCD	Effect of the mean HRL Tree Cover Density	−0.03
		(−0.38, 0.31)
mean_GHSL	Effect of the mean Global Human Settlement Density	0.21
		(−0.14, 0.69)
ai_mean	Effect of the mean aggregation index	−0.14
		(−0.49, 0.19)
mean_slope	Effect of the mean slope	−0.20
		(−0.56, 0.15)
time_of_daynight	Effect of night as the time of day	−0.08
		(−0.74, 0.55)
min_distance_to_road_scaled	Effect of the scaled minimum distance from the resting‐site centroid to roads	0.07
		(−0.23, 0.40)
min_distance_to_river_scaled	Effect of the scaled minimum distance from the resting‐site centroid to rivers	−0.02
		(−0.32, 0.31)
dist_to_veg_scaled	Effect of the scaled minimum distance from the resting‐site centroid to plant cover	−0.20
		(−0.58, 0.20)
dist_to_building_scaled	Effect of the scaled minimum distance from the resting‐site centroid to buildings	0.10
		(−0.22, 0.43)

*Note:* The 95% highest posterior density intervals (HPDIs) are shown in brackets. No covariate had a considerable effect.

**FIGURE 8:**
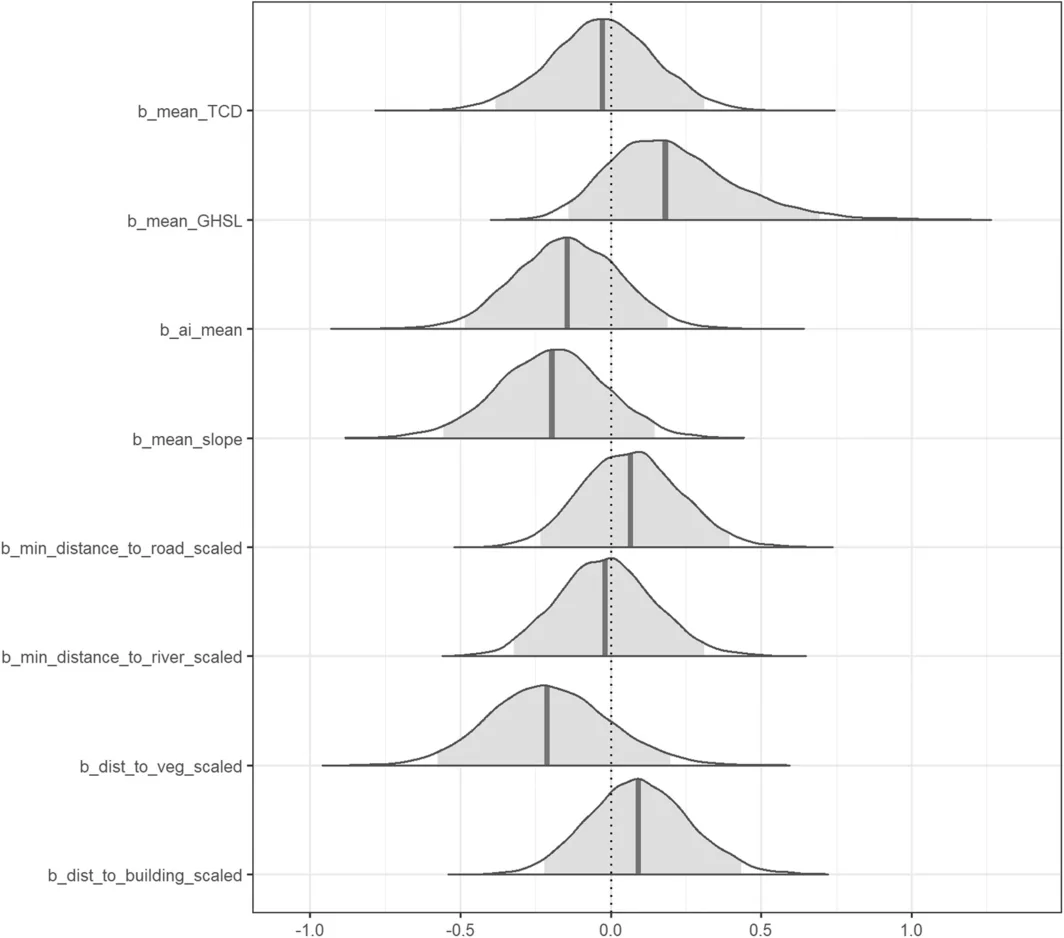
Posterior effects of the continuous predicting variables estimated by the Bayesian gamma regression model with turning angle as the dependent variable. Model estimates (marked with a vertical bold grey line) and HPDIs (marked in grey) are presented on the logarithmic scale. Effects are considered considerable when the HPDI does not include zero.

### 3.3 Reproductive Confirmation

In spring 2024, camera‐trap data from a mid‐elevation montane site situated at 1230 m a.s.l. confirmed the presence of a second individual and the subsequent birth of two pups, most likely in late April. Photographic evidence of the family interacting at multiple sites suggests the establishment of a family group (Video 1). Because the collared male remained resident in the territory before and after the camera‐trap observations, the most parsimonious explanation is successful settlement followed by local reproduction.

**VIDEO 1:** Camera‐trap footage recorded in spring 2024 in the newly established territory of the studied golden jackal. Video content can be viewed at https://onlinelibrary.wiley.com/doi/10.1002/ece3.74511.

## 4 Discussion

This study documents an 11‐day dispersal event of a subadult male golden jackal, culminating in successful settlement and reproduction in a sub‐alpine environment. To our knowledge, this represents one of the first fine‐scale demonstrations of a golden jackal's ability to traverse mountainous terrain, repeatedly cross major highways, and establish a stable home range above 1000 m a.s.l. Although previous studies have focused on natal dispersal of golden jackals (Bogdanowicz et al. 2025; Lanszki et al. 2018) or on small‐scale movement within their home ranges (Csányi et al. 2025; Fenton et al. 2021), our study focused on the fine‐scale movement of one individual and conducted in‐depth analyses of its dispersal trajectory.

Visual evaluation of the individual's movement patterns revealed a dispersal event in November 2023 after an initial period of exploratory behaviour. The dispersal process was characterised by successful navigation of both anthropogenic and natural barriers in a subalpine environment. Dispersal ended with settlement in a permanent territory at the entrance to the Malta Valley, Austria.

The characteristics of the dispersing jackal are in line with those of previous studies. Subadults are the age group that has been shown to disperse most frequently in an experimental study by Kapota et al. (2016). Additionally, the golden jackal monitored by Lanszki et al. (2018) during natal dispersal in Hungary, a collared male that dispersed from western Slovenia to coastal Croatia (Potočnik et al. 2019), and two collared jackals dispersing in Greece (unpublished data) (Giannatos 2004) were all subadults.

We found that the dispersal event lasted approximately 11 days. After settlement, the golden jackal sporadically left its newly established home range during exploratory movements. The recorded dispersal duration aligns with findings in previous studies. Lanszki et al. (2018) have reported a dispersal transience duration of 12 days. Meanwhile, Potočnik et al. (2022) observed dispersal between 8 and 97 days in six collared golden jackals aged between seven and 24 months.

Moreover, the movement distances of golden jackals within their home ranges vary throughout the year, and their movements may even exceed the boundaries of their home ranges (Csányi et al. 2025; Pantelić et al. 2024). Seasonal movement patterns in golden jackals are largely determined by reproductive events such as mating, pregnancy and pup‐rearing (Csányi et al. 2025; Pantelić et al. 2024). Indeed, the exploratory movements observed in our study occurred in February and coincided with the golden jackal mating season (January–March). Although Csányi et al. (2025) stated that values for home ranges are usually relatively small during this period, we believe that these excursions likely had a purpose to increase the chances of the studied jackal finding a mate. Extra‐territorial excursions during the mating season have been reported in Eurasian lynx (Liang et al. 2026), European badgers (Kelly et al. 2020), and suggested in grey wolves (Mech 2020).

The lack of environmental covariates that had an effect on the jackal's selection of resting sites is striking, as we expected that resting sites would be preferred in heterogeneous areas with sufficient amounts of cover, food and water. Indeed, the availability of these factors has been identified as a crucial determinant of habitat suitability for golden jackals. Kamler et al. (2021) have identified the availability of herbaceous cover, water, and prey as the main determinants of territory size in golden jackal groups in Cambodia. Natural vegetated areas that provide cover have similarly been described as important factors in habitat selection by golden jackals (Frangini et al. 2022), especially during the day (Fenton et al. 2021). Moreover, Torretta et al. (2023) observed that dispersing wolves primarily selected resting sites away from anthropogenic activity in both time and space. Our findings can be explained by the considerable individual variation in habitat and resting‐site selection reported in golden jackals and wolves (Fenton et al. 2021; Frangini et al. 2022; Torretta et al. 2023). Due to their ecological plasticity, some individuals may be less affected by anthropogenic activity, especially since human presence often correlates with greater food availability (Csányi et al. 2025; Frangini et al. 2022; Fenton et al. 2021). Furthermore, although resting site selection and habitat selection are generally correlated (Frangini et al. 2022), factors influencing habitat selection in golden jackals do not necessarily have the same effect on resting site selection, as resting sites are not destined to serve as long‐term habitats.

As expected, both nighttime and the degree of landscape fragmentation positively affected the jackal's step length. These patterns reflect a tendency to move more extensively during the night and in homogeneous areas, and they may represent a temporal avoidance of high risk situations as well as less interrupted movement in more uniform landscapes, underlining the tendency of the jackal to avoid human activity during the day and to quickly transit through the area, as has been documented in wolves (Mech 2020; Morales‐González et al. 2022). Torretta et al. (2023) observed similar patterns in three GPS‐collared wolves, all of which showed greater step lengths at night than during the day, and two of the three showed greater step lengths in landscapes deemed highly unsuitable. Similarly, for golden jackals, numerous studies have shown higher movement rates at night, confirming a strong temporal avoidance of golden jackals towards anthropogenic activity (e.g., Fenton et al. 2021; Lanszki et al. 2018; Pantelić et al. 2024).

Matching our predictions, the jackal's movement persistence and direction were not affected by any characteristics related to mountainous landscapes (e.g., slope, elevation). Furthermore, potential physical barriers such as roads, rivers, and even human settlements did not influence the dispersing jackal's movement. This aligns with previous findings, where golden jackals have demonstrated their ability to disperse across heterogeneous landscapes and to overcome both natural and anthropogenic movement barriers such as motorways (Lanszki et al. 2018; Potočnik et al. 2022). After the dispersal, the golden jackal successfully established a stable reproductive group, marking this the first documented case of a golden jackal settling in this subalpine region.

Our study focused on the dispersal of a single individual, through which we have gained valuable new insights at a high resolution; however, it remains challenging to extend our findings to the population level. Moreover, due to technical limitations, we were unable to record telemetry data at regular intervals and were therefore required to interpolate our dataset. Because we were unable to retrieve the GPS collar containing additional data, we have only limited information regarding the events following the successful settlement and reproduction. Consequently, statistical inferences should be interpreted as individual‐level patterns rather than population‐level effects.

Future research should focus on building a larger database by including more golden jackal individuals and using regular sampling intervals. Furthermore, it will be valuable to integrate genetic analyses to determine the origins and connectivity of newly established (sub)alpine populations. Additionally, a broader‐scale assessment of golden jackal movement patterns across Central Europe would provide a more comprehensive understanding of the species' ongoing expansion. Given the increasing presence of golden jackals in transboundary regions, international collaboration remains crucial for effective monitoring and management. Our study, therefore, further underscores the need for transboundary collaboration in monitoring and managing expanding carnivore populations. Finally, the establishment of a breeding pair in this subalpine region underscores the importance of long‐term monitoring to assess population dynamics, potential human–wildlife conflicts and ecological interactions with native fauna.

## 5 Conclusion

This study provides the first detailed behavioural reconstruction of a golden jackal dispersing into and settling within a subalpine environment. Its movements demonstrate that at least some individuals are capable of traversing mountainous terrain and major infrastructure. Step length analysis indicated longer movements at night and in more aggregated, homogeneous landscapes, suggesting that both temporal and landscape characteristics may influence movement behaviour during dispersal. The subsequent successful settlement and reproduction of this golden jackal in Carinthia (Austria) suggests that suitable habitat exists even in regions traditionally considered marginal for jackal survival. These findings highlight the species' behavioural flexibility and help explain the rapid and ongoing expansion of golden jackals across Europe. Expansion into novel environments, including (sub)alpine regions, raises important ecological and management questions.

## Author Contributions

**Jonas Custers:** conceptualization (equal), data curation (lead), formal analysis (lead), methodology (lead), visualization (equal), writing – original draft (lead), writing – review and editing (supporting). **Jennifer Hatlauf:** conceptualization (supporting), funding acquisition (lead), project administration (lead), supervision (lead), validation (supporting), writing – original draft (supporting), writing – review and editing (supporting). **Ancolie de Bergeyck:** formal analysis (equal), methodology (equal), writing – review and editing (equal). **Jaka Črtalič:** validation (supporting), writing – review and editing (supporting). **Boštjan Pokorny:** validation (supporting), writing – review and editing (supporting). **Hubert Potočnik:** funding acquisition (lead), project administration (equal), supervision (equal), writing – review and editing (equal).

## Funding

This work was supported by BOKU University, Open Access Funding and Slovenian Research and Innovation Agency (ARIS), P1‐0184, P4‐0107, V1‐0231 and Ministry of Agriculture, Forestry and Food of the Republic of Slovenia: V1‐0231.

## Conflicts of Interest

The authors declare no conflicts of interest.

## Supporting information


**Figure S1:** ACF plots representing residuals from the Bayesian models with continuous responses.

**Figure S2:** Plots representing the distance‐elevation travel trajectory of the studied golden jackal during the dispersal process, showing (a) selected resting sites, (b) step length (m/2 h) and (c) turning angle (radians). Road and water crossings are marked with grey squares and blue dots, respectively.

**Figure S3:** Isopleth area curves showing (a) the area of each isopleth for different values of *k* with *s* = 0.02 and (b) the ratio of edge length (i.e., total perimeter) to area for each isopleth level for different values of *k* with *s* = 0.02.

**Data S1:** WAMBS checklist applied to the Bayesian model for resting‐site selection.

**Data S2:** WAMBS checklist applied to the Bayesian model for step length.

**Data S3:** WAMBS checklist applied to the Bayesian model for turning angle.

## Data Availability

The data that support the findings of this study are openly available in OSF at https://osf.io/kcbft/overview?view_only=0506840894cd4eeb879e0839fe0a7e06.
