# Supplementary material for: Overcoming Alpine Barriers: The First Documented Case of Golden Jackal (Canis aureus) Settlement in a Subalpine Environment
Source: Ecol Evol. 2026 Oct 23;16(10):e74511. doi: 10.1002/ece3.74511 (PMC13691633; doi:10.1002/ece3.74511)
Supplement: Supplementary file 1 [file ECE3-16-e74511-s002.pdf]

POINT 1: Priors

Flat priors were used.

POINT 2: Chain convergence (fat 'catterpillar' = ok)

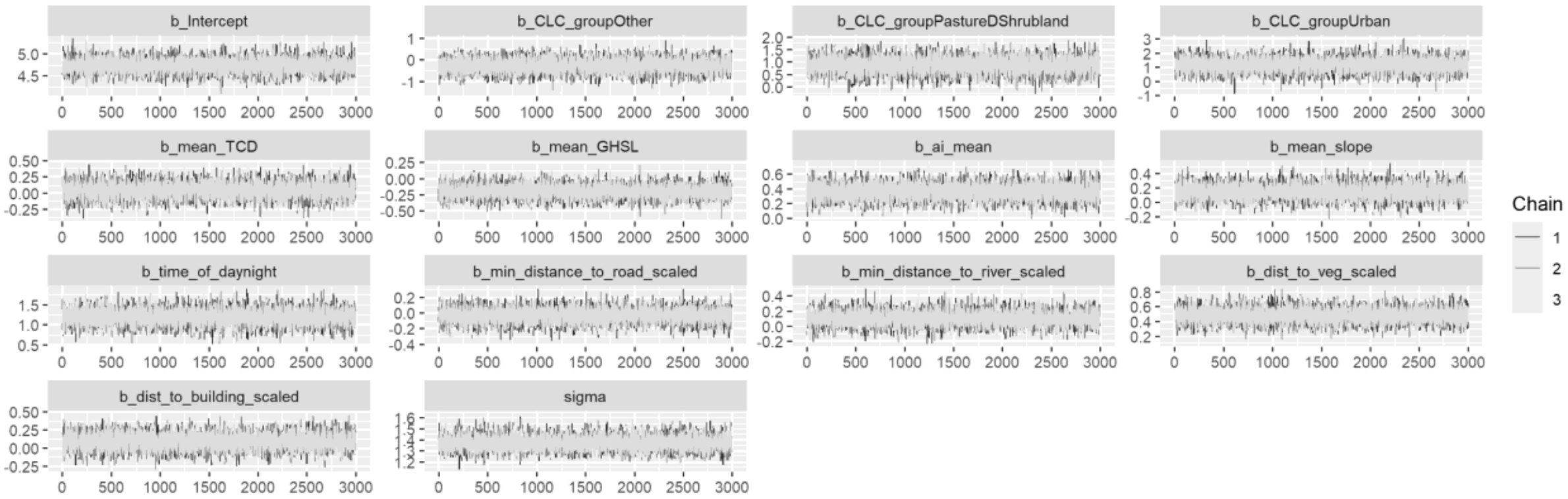

POINT 3: Does convergence remain after doubling the number of iterations?

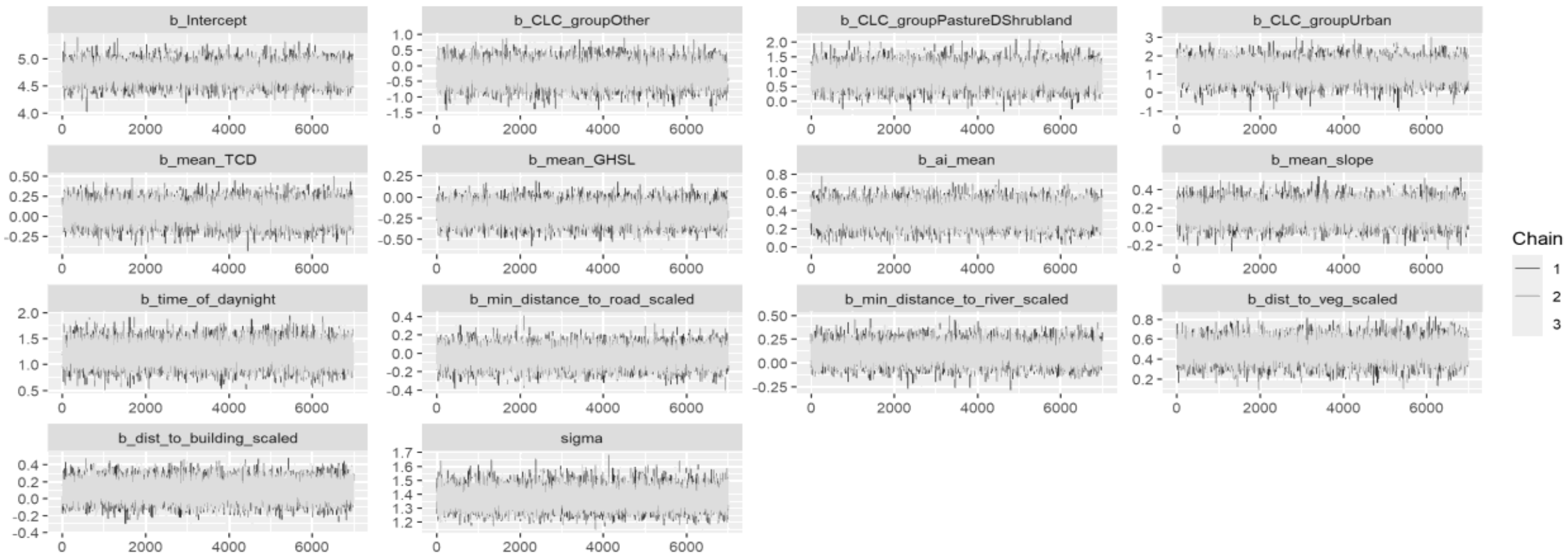

POINT 4: Does the posterior distribution histogram have enough information? (histograms look smooth and have no gaps or other abnormalities = ok, they do not have to be symmetrical)

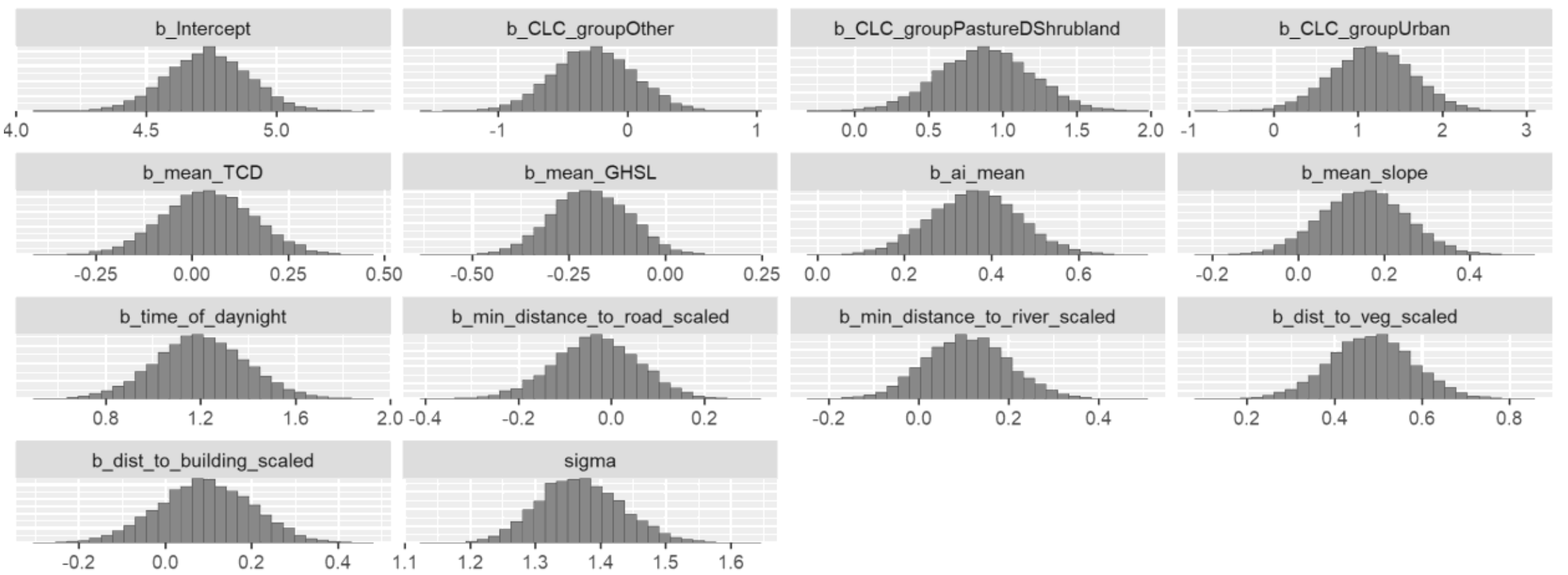

POINT 5: Do the chains exhibit a strong degree of autocorrelation?

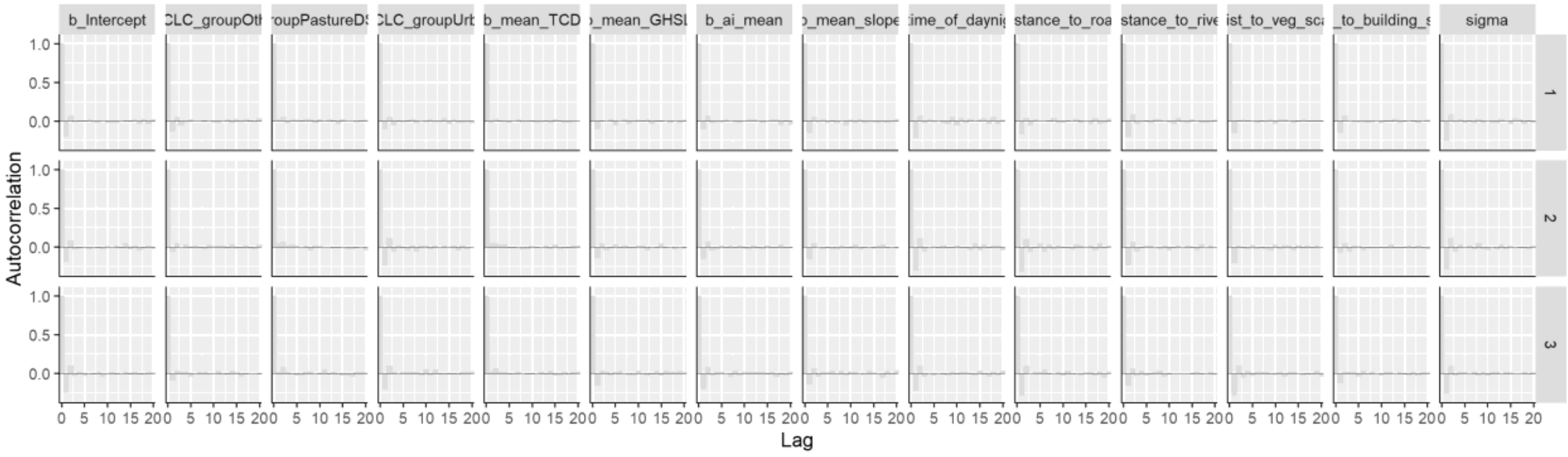

POINT 6: Do the posterior distributions make substantive sense?

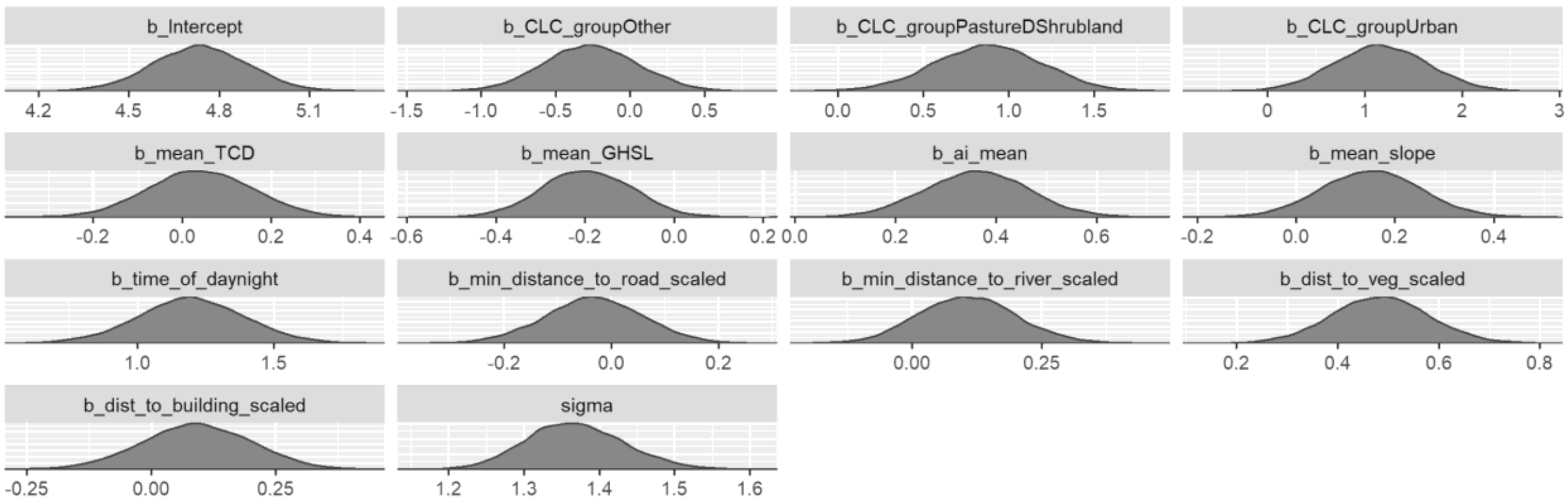

POINT 7: Do different specifications of the variance priors influence the results?

Not applicable, no variance level priors specified.

POINT 8: Is there a notable effect of the prior when compared with non-informative priors?

Flat priors were already used.

POINT 9: Are the results stable from a sensitivity analysis?

Stable results, magnitude and direction don't change when changing priors.
